# Structural analysis of antigenic variation and adaptive evolution of the H5N1 neuraminidase gene

**DOI:** 10.1371/journal.pcbi.1013903

**Published:** 2026-01-16

**Authors:** Muyiwa S. Adegbaju, Oluwabuyikunmi Owo-Odusi, Eden T. Wirtz, Olanrewaju B. Morenikeji, Olusola Ojurongbe, Bolaji N. Thomas

**Affiliations:** 1 Department of Biomedical Sciences, Rochester Institute of Technology, Rochester, New York, United States of America; 2 Department of Biology, College of Natural and Health Sciences, Virginia State University, Petersburg, Virginia, United States of America; 3 Department of Medical Microbiology and Parasitology, Ladoke Akintola University of Technology, Ogbomosho, Nigeria; 4 Center for Emerging and Re-emerging Infectious Diseases (CERID), Humboldt-Bayer Foundation Research Hub, Ladoke Akintola University of Technology, Ogbomosho, Nigeria; Hebrew University of Jerusalem, ISRAEL

## Abstract

The concern regarding H5N1 outbreak, particularly the accelerated mutagenesis of its core genomic elements, underscores the persistent threat of influenza to global health. Neuraminidase (NA), a pivotal sialidase integral to virion egress and propagation, comprises nine distinct isoforms, exhibiting unique evolutionary trajectories and structural adaptations. Despite extensive characterization of hemagglutinin subtypes, the functional divergence of the nine NA subtypes remains inadequately understood. To address this gap, we conducted a structural analysis of NA subtypes, employing structural superimposition and motif-guided sequence alignment to delineate subtype-specific residues. Hierarchical clustering stratified the nine NA subtypes into four distinct subgroups: NA2 (subgroup I), NA1 and NA4 (subgroup II), NA9/NA7/NA6/NA3 (subgroup III), and NA8/NA5 (subgroup 4). We identified 40 highly conserved and functionally significant amino acid loci, likely modulating enzymatic activity and substrate specificity across subtypes. To investigate the structural basis of adaptation in H5N1, we generated NA1 mutants by swapping family specific position (FSP) residues and analyzed their dynamics using Molecular Dynamics (MD) simulations, complemented by a deep phylogenetic analysis across six host reservoirs. MD simulation parameters reveal a dynamic paradox: the Wild-Type (WT) NA1 maintains a conserved global compactness Rg, which masks a complex, bi-modal switching mechanism essential for its catalytic function, validated by multi-basin free energy landscape (FEL) topography. We identify Lysine-207 (K207) as the master determinant of this switching mechanism and the enzyme’s dynamic fate. Substitutions at this conserved nexus produced diametrically opposite outcomes: K207W imposed structural rigidification (abolishing the switch), K207H achieved dynamic preservation, and K207I drove expanded disorder and collapse. Furthermore, dynamic correlation analysis shows that these single-point substitutions function as molecular switches that significantly re-wire the enzyme’s allosteric communication networks, extending far beyond the active site. To assess the role of NA1 in host tropism and adaptive evolution, we conducted a phylogenetic analysis of *NA1* genes from H5N1 isolates across multiple host reservoirs; *H. sapiens*, *G. gallus*, *Anser anser domesticus*, *M. gallopavo*, *B. taurus*, and *C. olor*. Notably, we observed opposing selection pressures and diversification patterns: *G. gallus* isolates showed signatures of positive selection consistent with hyper-reassortment, while human isolates displayed highly diverse, sporadic spillover events. We conclude that the evolutionary contribution of NA1 to H5N1 host adaptation is not encoded in static structure, but certain residues such as K207 defines a pivotal mechanism for regulating the enzyme’s function through dynamic states. Our MD data thus proposes a novel strategy for next-generation antivirals by targeting this dynamic vulnerability—the Nexus for Dynamic Ablation—to permanently entrain the enzyme in a non-functional conformation.

## Author summary

The persistent threat of H5N1 influenza demands a deeper understanding of its critical enzyme, Neuraminidase (NA). We first conducted a structural analysis of all nine NA subtypes, stratifying them into four distinct phylogenetic subgroups and identifying 40 highly conserved functional residues. Focusing on the H5N1 NA1, Molecular Dynamics simulations revealed a dynamic paradox: while the enzyme maintains conserved global compactness, its catalytic function is regulated by a complex, bi-modal switching mechanism. We identified Lysine-207 (K207) as the master determinant of this dynamic fate. Substitutions at K207 produced diametrically opposite outcomes—from structural rigidification to expanded disorder—and significantly re-wired the enzyme’s allosteric communication networks far beyond the active site. Phylogenetic analysis of NA1 across host reservoirs further showed that NA1’s adaptive evolution is defined by these dynamic states, not static structure. Our data proposes a novel strategy for next-generation antivirals by targeting this dynamic vulnerability—the Nexus for Dynamic Ablation—to permanently lock the enzyme into a non-functional state.

## Introduction

Highly pathogenic avian influenza A (HPAI) H5N1 viruses continue to pose a significant threat to global health due to their high virulence and persistent pandemic potential [1]. Effective surveillance and a deep understanding of viral adaptation are crucial for mitigating the risk of animal-to-human or human-to-human transmission and severe disease. A key determinant of influenza virus pathogenicity and a primary target for host immune responses and antiviral interventions is the surface glycoprotein neuraminidase (NA) [2,3]. NA facilitates viral release from infected cells and is considered a promising vaccine target, although its inherent variability complicates vaccine development [4–6].

Influenza A viruses are classified into 11 distinct neuraminidase subtypes (N1-N11), based on their antigenic properties. The structural flexibility of NA significantly contributes to influenza virus’ adaptation to diverse hosts, highlighting a key facet of molecular evolution. NA’s capacity for conformational changes, particularly in regions such as the ‘150 loop,’ directly impacts its enzymatic activity, influencing viral replication, transmissibility, and drug susceptibility [7]. Early molecular dynamic simulations (MDSs) on avian influenza N1, initiated from apo- and oseltamivir-bound crystal structures (PDB ID: 2HTY, 2HU0), demonstrated the 150-loop’s ability to sample conformations significantly wider than any previously observed crystal structures (including both open and closed forms). These MDS also suggested a rapid loop switching motion coupled to the neighboring 430-loop, which further expands the active site cavity. This dynamic behavior of the 150-loop has been recognized as a potential opportunity for novel drug design [7]. This flexibility is also essential for maintaining a functional balance with hemagglutinin (HA), a critical determinant for successful viral replication and host specificity [8]. Given that protein flexibility is essential for its biological functions, including protein-nucleic acid or protein-protein interactions and ligand binding [9,10], NA presents a compelling model for studying the interplay between protein flexibility, functional diversity, and evolvability, within the broader context of molecular evolution and its ultimate role in influenza host adaptation. Within this diverse landscape of NA subtypes, our study specifically focuses on the nine major neuraminidase subtypes (N1-N9), which are predominantly of avian origin and exhibit distinct structural characteristics from N10 and N11 [11–15].

Protein evolution often involves subtle structural changes due to accumulating mutations, leading to distinct functional properties. In this context, “family-specific positions” (FSPs) are crucial, as they exhibit variability across enzyme subtypes yet remain conserved within a given subtype [16], driving functional diversification through modulation of enzymatic properties and protein flexibility [17–20]. The H5N1 *NA1*, exhibits high genetic plasticity, undergoing continuous antigenic drift and, less frequently, antigenic shift [21]. However, the rapid mutation pace of H5N1 is a cause for concern, as it could evolve into a deadly human virus with only a slight change in receptor usage [1,2]. For example, laboratory studies have demonstrated that a single engineered mutation in the Hemagglutinin (HA) protein of bovine influenza H5N1 can shift its specificity to human receptors [22], illustrating a potential pathway for host adaptation. This inherent capacity for adaptive evolution, driven by mutations in various viral proteins, is a constant concern. Such mutations enable the virus to evade pre-existing host immunity (often driven by changes in key antigenic sites) or contribute to the emergence of antiviral resistance (notably against neuraminidase inhibitors). Furthermore, adaptive mutations can facilitate adaptation to new hosts, as observed in recent outbreaks in cattle, where polymerase mutations have been identified as key early adaptations [23]. Given NA1’s critical role in viral release and its own inherent variability, understanding its evolution across diverse hosts (avian and mammalian) is equally critical for predicting viral adaptation and potential zoonotic spillover events. While the NA active site is highly conserved, mutations at other FSPs can still impact antigenicity, receptor binding, and drug susceptibility through allosteric effects or altered protein dynamics. However, even with the study focusing on regions like the well-characterized 150-loop [7], a comprehensive understanding of the molecular mechanisms by which specific FSPs drive both inter-subtype functional diversity across N1-N9 and intra-subtype host adaptation in H5N1 NA1, particularly those FSPs beyond the 150-loop, remain underexplored at an atomic level.

**Fig 1:**
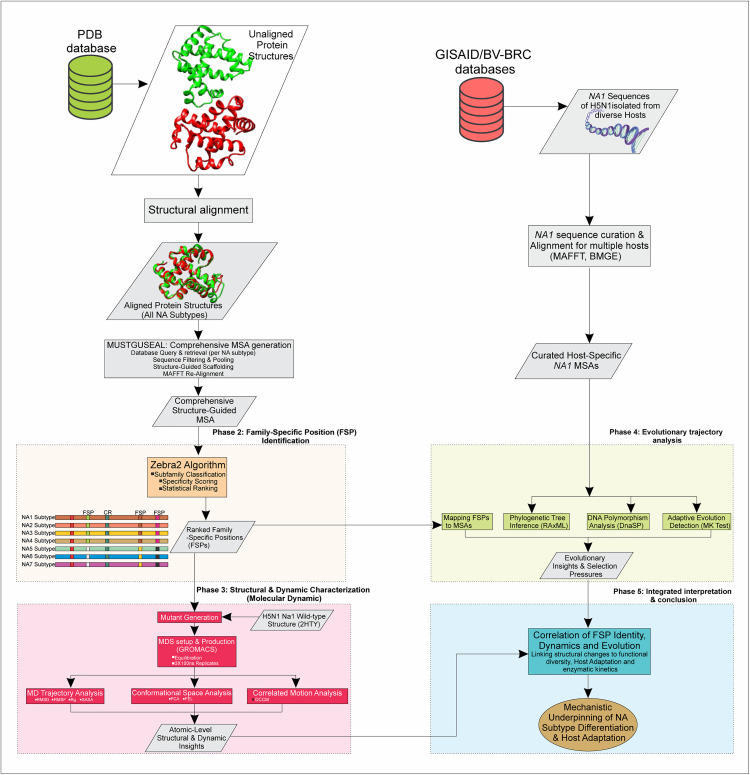
Computational pipeline for structural and evolutionary analysis of H5N1 neuraminidase (NA1) and subtype-specific positions. This figure presents the multi-phase computational pipeline used in the study. Symbols represent data and processes: cylinders indicate databases (green for PDB structural data, red for GISAID/BV-BRC sequence data), parallelograms denote data inputs/outputs (grey for raw sequences and processed data), and rectangles represent processing steps colored by phase. Dashed boxes group elements by phase, and ovals show final outcomes. Key visual elements include 3D protein structures to illustrate alignment, colored bars for Family-Specific Positions and conserved residues and DNA helix structure to illustrate *NA1* from multiple hosts.

To address this critical knowledge gap, we employed a novel, integrated computational approach (Fig 1). We first utilized the Zebra 2 algorithm to identify and statistically rank FSPs across the nine major NA subtypes through comparative structural analyses and superimposition, thereby identifying positions conserved among subfamilies and those peculiar to specific subgroups, linking them to functional diversity across these subtypes. Subsequently, we investigated the structural and dynamic consequences of these highly ranked FSPs in H5N1 NA1 (wild type and mutated versions) using MDSs, providing atomic-level insights into protein dynamics, conformational changes, and the impact of these mutations on structural flexibility and function. Furthermore, we characterized the evolutionary trajectories of these FSPs by quantifying synonymous versus nonsynonymous substitution rates across H5N1 isolates from a spectrum of avian and mammalian hosts. This multifaceted approach aims to establish direct correlations between residue identity, alterations in enzymatic kinetics, allosteric regulation, and viral replicative fitness, thereby revealing the mechanistic underpinnings of subtype differentiation and host adaptation. By providing detailed structural and dynamic insights into H5N1 NA1 evolution and the broader functional diversity of NA subtypes, this work will enhance our understanding of viral immune evasion and antiviral resistance mechanisms. The findings hold significant implications for the rational design of more effective influenza vaccines and the development of robust antiviral strategies, thereby contributing to global pandemic preparedness efforts.

## Results

### Structural analysis

**Fig 2:**
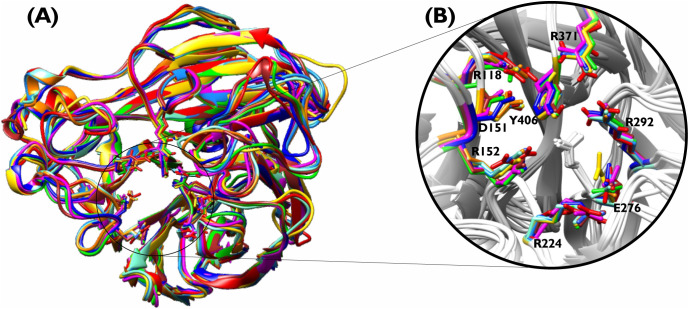
Structural superimposition of nine avian influenza A neuraminidase (NA) subtypes (N1–N9) illustrating conserved and variable regions essential for identifying Family-Specific Positions (FSPs). (A) Shows the overlay of NA subtypes as cartoons highlighting shared folds and subtype-specific conformational differences. Structures are color-coded for clarity and as follows; 2HTY (Beige/Tan), 1NN2 (Sky Blue), 4HZY (Salmon or Light Coral), 2HTV (Light Green), 3SAL (Golden Yellow/Amber), 4QN4 (Deep Pink/ Fuchsia), 4QN3 (Light grey), 2HT5 (Pale Magenta), and 7NN9 (deep blue). (B) Zooms in on the conserved active site and 150-loop residues (labeled by 2HTY numbering), demonstrating their critical and consistent roles across all subtypes, which supports functional analysis and FSP identification via the ZEBRA2 algorithm.

The neuraminidase protein typically functions as a homotetramer, with its catalytic domain (spanning residues 83–468) possessing a well-resolved crystal structure crucial for enzyme activity. This domain was the focus of our structural analysis. We examined the structural relationships among the nine avian influenza A virus (IAV) NA subtypes by selecting a single monomeric chain (Chain A) of NA1 (PDB: 2HTY [24]) as the reference structure for superimposition. This reference was then superimposed with selected chains from other NA subtypes, including 1NN2 (NA2: chain A [25]), 4HZY (NA3: chain A [26]), 2HTV (NA4: chain B [24]), 3SAL (NA5: chain B [27]), 4QN4 (NA6: chain B [28]), 4QN3 (NA7: chain B [28]), 2HT5 (NA8: chain A [24]), and 7NN9 (NA9: chain A [29]) (Fig 2A). This superimposition revealed a high degree of overall structural conservation among the subtypes, characterized by a shared catalytic domain fold, alongside distinct regions of conformational variability. Sequence identities among these subtypes ranged from 50% to 70.8% (Table 1). Further examination of the catalytic site (Fig 2B) revealed that key substrate-contacting residues are not only highly conserved in sequence but also maintain remarkably similar spatial positioning across all nine NA subtypes. These spatially conserved residues, displayed as sticks and labeled with 2HTY numbering, include essential components of the active site (R118, E276, R292, R371, Y406, R224) and the flexible 150-loop (D151, R152).

**Table 1:** Pairwise sequence identity of monomeric chains from nine avian influenza neuraminidase subtypes used for structural superimposition.

	2HTY	1NN2	2HT5	2HTV	4HZY	4QN3	4QN4	3SAL	7NN9
**2HTY**									
**1NN2**	44.70								
**2HT5**	55.81	45.36							
**2HTV**	70.80	43.81	58.46						
**4HZY**	45.99	51.80	45.10	45.36					
**4QN3**	44.96	48.45	43.59	43.59	45.88				
**4QN4**	47.03	50.52	47.18	43.85	48.20	62.31			
**3SAL**	56.85	42.01	69.23	56.67	43.81	43.59	45.27		
**7NN9**	47.70	47.68	42.01	42.01	46.91	61.60	69.07	41.49	

(PDB IDs and corresponding NA subtypes: 2HTY (NA1), 1NN2 (NA2), 4HZY (NA3), 2HTV (NA4), 3SAL (NA5), 4QN4 (NA6), 4QN3 (NA7), 2HT5 (NA8), and 7NN9 (NA9)).

**Fig 3:**
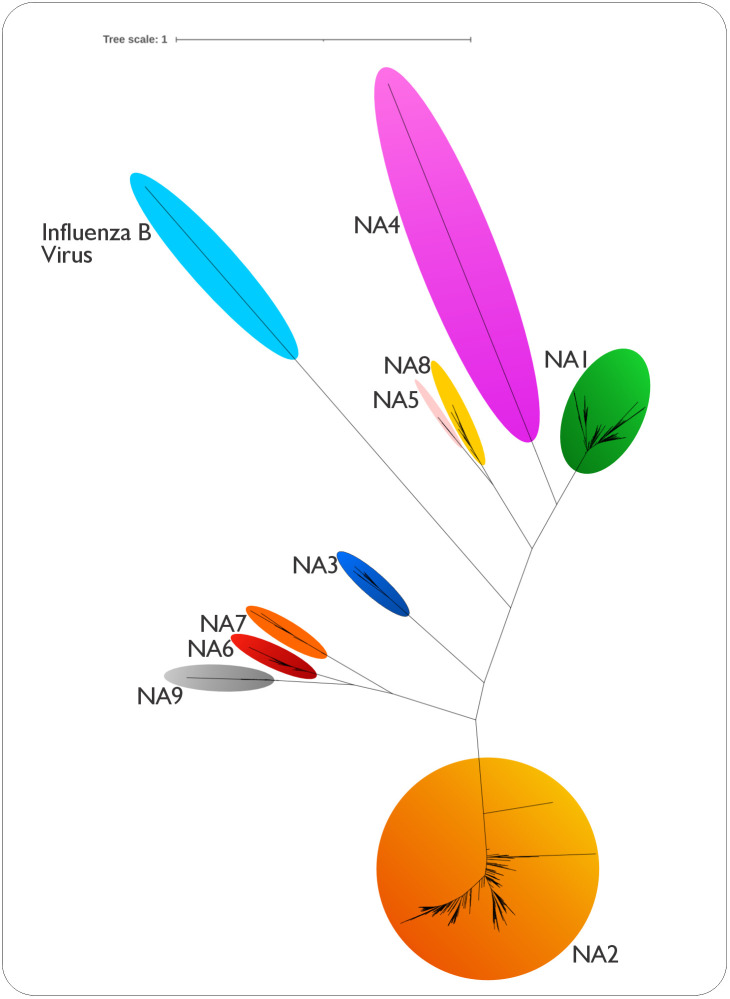
Phylogenetic reconstruction of nine influenza A neuraminidase subtypes. This unrooted phylogenetic tree reconstructs the evolutionary relationships among 645 neuraminidase homologs, representing the nine major influenza A virus (IAV) NA subtypes (N1-N9). Inferred using PhyML + SMS version 3.0 based on the comprehensive structure-guided alignment (as detailed in Fig 1) with SH-like aLRT branch support, the tree distinctly groups the NA subtypes into their nine established clades. These clades further cluster into two broad phylogenetic groups: Group 1 (N1, N4, N5, N8) and Group 2 (N2, N3, N6, N7, N9). Notably, a distinct node representing Neuraminidase from Influenza B virus is positioned between these two major Influenza A groups, reflecting its evolutionary divergence. Each NA subtype is visually differentiated by uniquely colored eclipse-shaped nodes.

The consistent positioning and chemical nature of these residues across diverse NA subtypes were observed, reflecting their critical role in maintaining core enzymatic function. Following a comprehensive structure-guided sequence alignment of 655 nonredundant NA homologs, an unrooted phylogenetic tree was constructed. This tree distinctly grouped IAV’s neuraminidases into nine subtypes, with NA1 exhibiting higher evolutionary affinity to NA4, NA8, and NA6 (Fig 3).

### Classification and sub-family specific positions

The comprehensive structure-guided alignment generated using MUSTGUSEAL (as detailed in our computational pipeline, Fig 1), comprised 645 neuraminidase homologs (see S1–S4 Figs), representing the diversity across all nine NA subtypes and their pooled sequences from databases. From this alignment, an unrooted phylogenetic tree was constructed (Fig 3). This phylogenetic analysis distinctly grouped IAV’s neuraminidases into their nine established subtypes, which further clustered into two broad phylogenetic groups (Group 1: N1, N4, N5, N8; Group 2: N2, N3, N6, N7, N9). Subsequently, this same comprehensive structure-guided alignment was analyzed with Zebra2. This analysis classified the 645 neuraminidase homologs into four statistically significant subgroups (Table 2), providing a more granular resolution compared to the two broad groups identified by the phylogenetic tree. The Zebra2 analysis, based on subfamily-specific residue positions, identified 100 invariant residues. These included specific active site residues (e.g., D131 and Y382 in NA1 numbering), along with 12 other conserved residues located at substrate recognition and Ca² ⁺ coordination sites.

**Table 2:** Classification and distribution of neuraminidase homologs into four statistically significant subgroups by Zebra2.

Sub-type#	Representative protein structure (PDB code)	Molecular Chain(s)	Molecular name	Proteins
1	1NN2	A	NA2	288
2	2HTV	A, B	NA4	155
	2HTY	A, B, C, D, E	NA1	
3	7NN9	A	NA9	51
	4QN3	A, B	NA7	
	4QN4	A, B	NA6	
	4HZY	A, B	NA3	
4	2HT5	A	NA8	28
	3SAL	A, B	NA5	

**Fig 4:**
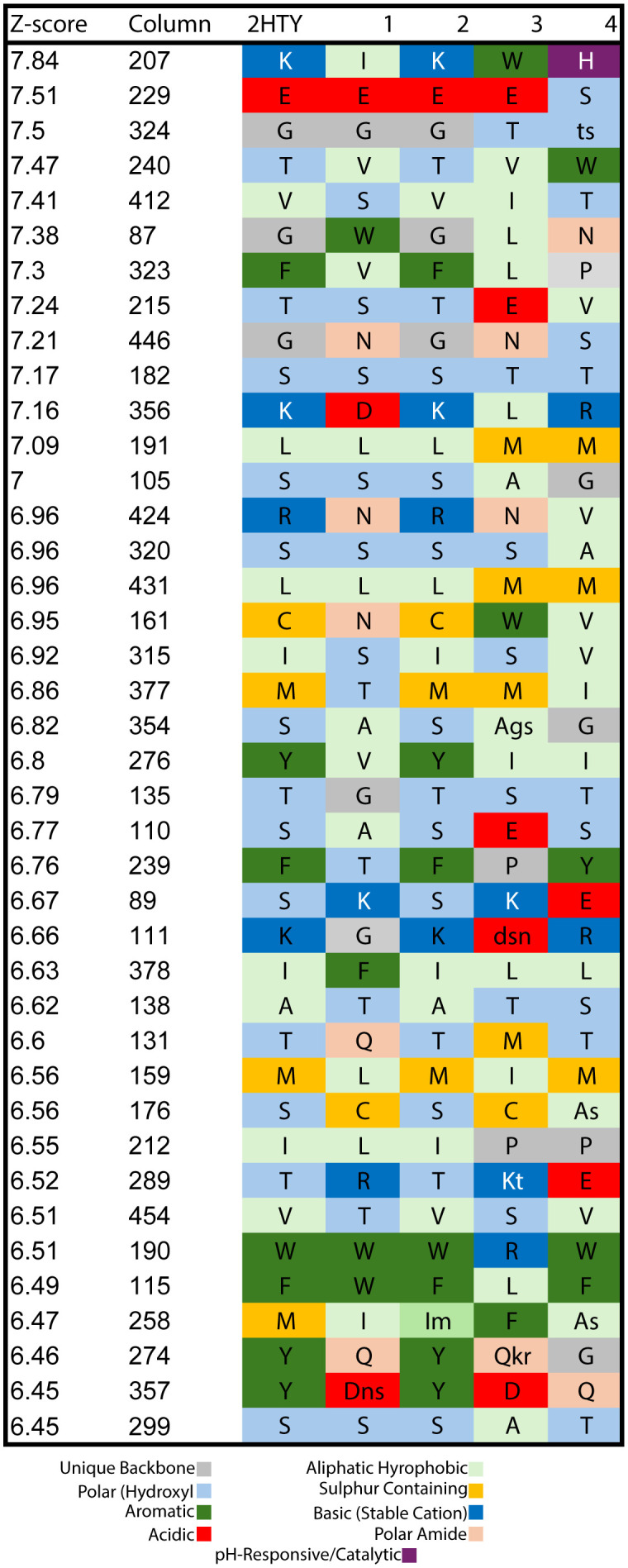
ZEBRA2-derived subfamily-specific position profile of neuraminidase homologs. This figure presents a ZEBRA2-generated subfamily-specific position profile (or conservation-variability profile), highlighting the top 41 statistically significant amino acid positions crucial for NA functional divergence across subtypes. Each row corresponds to a specific residue position from the comprehensive structure-guided alignment, with its Z-score (statistical significance of specificity) listed in descending order on the left, directly corresponding to its alignment position. Residue conservation is annotated by letter case: capital letters (>70% conservation) denote highly conserved residues, while lowercase letters (30-70% conservation) represent partially conserved. Where applicable, the three most frequent amino acids are listed. The figure includes an explicit, color-coded key that defines the residue identity based on nine distinct structural and functional groups for immediate interpretation. These groups are: Unique Backbone (G, P: Grey); Aliphatic Hydrophobic (A, V, L, I: light Green); Polar (Hydroxyl) (S, T: Light Blue); Sulfur-Containing (C, M: Deep Yellow); Aromatic (F, Y, W: Green); Basic (Stable Cation) (K, R: Deep Blue); Acidic (D, E: Red); Polar (Amide) (N, Q: Faded Pink); and Histidine (pH-Responsive/Catalytic) (H: purple). This profile directly visualizes the FSPs identified by ZEBRA2, pinpointing variations correlating with functional specialization within the NA protein family.

Furthermore, Zebra2 identified subfamily-specific positions with the highest Z-scores (Fig 4), indicating variations that correlate with functional specialization within the NA protein family.

### Comparative analysis of structural dynamics and solvent accessibility

**Fig 5:**
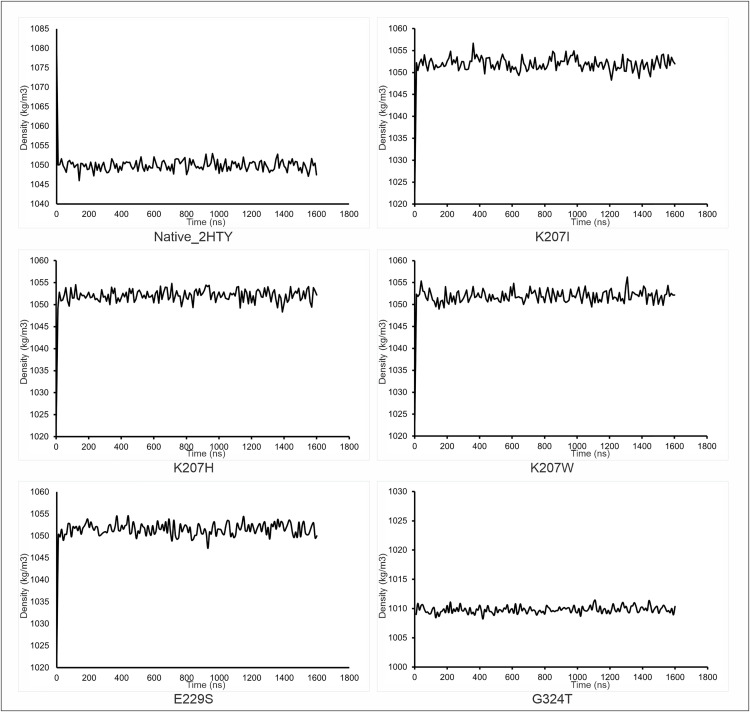
Molecular Dynamics Equilibration Trajectories for Native and Mutant Neuraminidase 1 (NA1) Constructs. This figure presents the equilibrium trajectories for the MD simulations of the Native NA1 structure (PDB ID: 2HTY) and the five investigated mutant constructs. Each panel plots the System Density (kg/m3) against the Simulation Time (ns) for the duration of the equilibration phase (1.6 ns). The stability of all trajectories, characterized by minimal fluctuations and the maintenance of a consistent average value over the 1600-time steps, confirms that all simulation systems achieved thermodynamic equilibrium prior to the production runs. This stability is a prerequisite for reliable statistical sampling of the conformational landscape and analysis of dynamic properties. The mutants shown are: K207I, K207H, K207W, E229S, and G324T.

**Fig 6:**
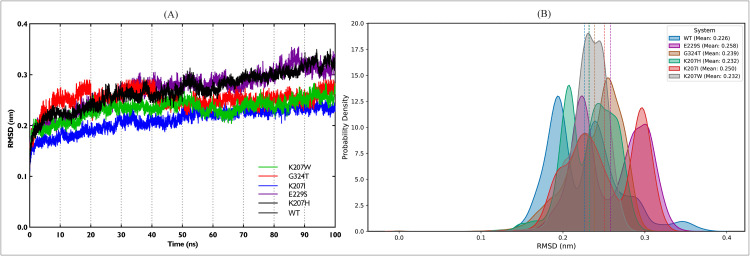
Cα RMSD Analysis: Time Evolution and Ensemble Distribution. The RMSD was calculated for the Cα atoms of the backbone relative to the energy-minimized structure of the WT protein. (A) Plots the RMSD (nm) over the first of the trajectory, illustrating the overall structural stability and convergence of the backbone. (B) Displays the KDE of the entire ensemble (300 ns total simulation). The KDE represents the probability density of sampling specific conformations, where the vertical dashed lines indicate the Mean for each system, visually confirming the statistically significant shifts in the ensemble (Table 3).

Using computational methods, NA1 mutant proteins were generated by swapping residues at the top three statistically significant FSPs identified by Zebra2 (Fig 4). Molecular dynamics simulations were performed for the wild-type and five mutant proteins to assess system stability. The Cα RMSD quantifies the time-dependent structural stability of the protein’s Cα-backbone relative to the starting energy-minimized fold. To ensure statistical certainty and fully characterize the equilibrium behavior, the production phase was executed using three independent replicas per system. The stability and non-drifting nature of the density plots for all systems confirmed successful equilibration (Fig 5). The three replicas for each system were verified for dynamic consistency (S1 Fig, confirming the convergence of each replicate) and then concatenated to form a single, aggregate trajectory. This full ensemble was used for all subsequent structural and dynamic analyses. The time evolution of the Cα-backbone for the WT and its five single-point mutants is presented in Fig 6A. All systems achieved dynamic equilibrium rapidly. The mutant exhibited the most substantial and highest sustained deviation, demonstrating the largest overall shift in structural coordinates. The ensemble data was used to calculate the Kernel Density Estimation (KDE), which maps the conformational sampling of the RMSD space (Fig 6B). The concatenated Probability Distribution Function (PDF) used for the KDE calculation is provided in S2 Fig. The Mann-Whitney test was applied to the RMSD ensemble data of the mutants versus the WT to confirm the statistical significance of the resulting structural shifts. As detailed in Table 3, the mean RMSD for all five mutants was found to be significantly higher than the ensemble (-value for all). This confirms that every substitution at positions 207, 229, and 324 causes a statistically robust shift away from the native fold.

**Table 3:** Statistical Significance for RMSD, R_g_, and SASA. P-values were calculated using the Mann-Whitney U test, comparing the full trajectory of the mutant system to the Wild-Type (WT) ensemble. The statistical significance threshold (α) was set at 0.05.

System	Mean	Median		P-value	P-value (vs WT)	
	RMSD (nm)	R_g_ (nm)	SASA (nm^2^)	RMSD	R_g_	SASA
**WT**	0.23	2.51	*160*	N/A	N/A	N/A
**E229S**	0.26	2.45	165	0	<10^-200^	<10^-200^
**G324T**	0.24	2.54	158	5.36e^-196^	<10^-200^	4.43e^-28^
**K207H**	0.23	2.47	159	1.68e^-262^	<10^-200^	2.27e^- 22^
**K207I**	0.25	2.53	164	0	<10^-200^	<10^-200^
**K207W**	0.23	2.50	161	1.73e^-222^	<10^-200^	<10^-200^

NB: Since the p-values for all mutants across all three parameters are consistently several orders of magnitude smaller than the highly stringent threshold, all mutants are confirmed to represent distinct conformational ensembles compared to the WT.

The RMSD derived from the ensemble precisely quantifies how each substitution re-engineers the native conformational landscape. The WT (Mean = 0.23 nm) system is characterized by a distinct bi-modal distribution. The K207H retains this crucial bi-modal characteristic, confirming that the inherent dynamic switching mechanism of the backbone is compatible with the Histidine substitution, albeit with altered peak positions. On the contrary, K207W displays a strictly unimodal distribution with the narrowest peak. The introduction of Tryptophan completely eliminates the native bi-modality, locking the backbone into a single, tightly defined, slightly expanded conformation. G324T (0.24 nm) also results in a unimodal distribution, demonstrating that the Glycine to Threonine substitution disrupts the native bi-modal switching. Crucially, its peak is significantly broader than the mutant, indicating a moderate degree of conformational heterogeneity—less disorder than, E229S, but more flexible than K207W. E229S (0.25 nm) and K207I (0.26 nm) mutants show the greatest rightward shift of the entire ensemble, confirming their preference for significantly expanded, less stable structures, as quantitatively supported by their highest mean values.

**Fig 7:**
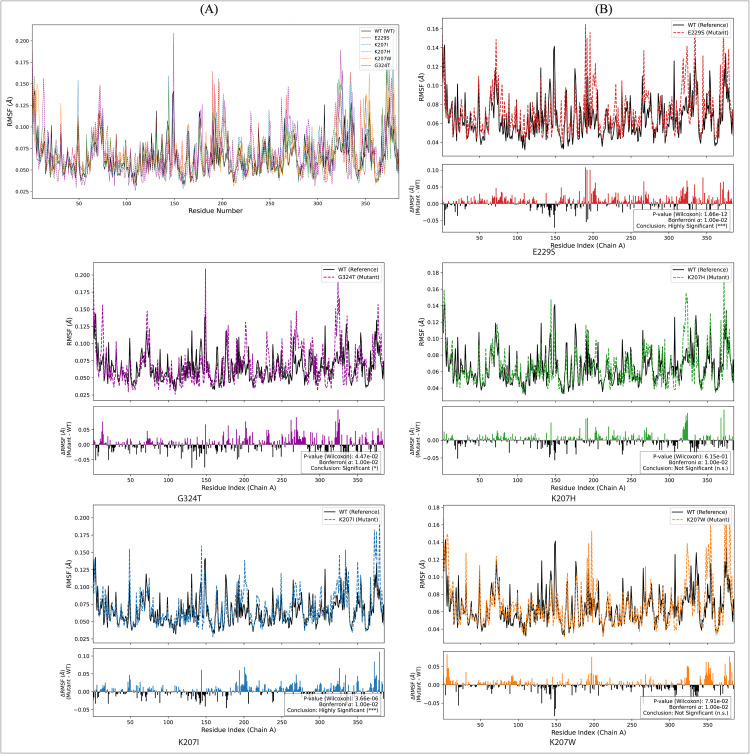
Root Mean Square Fluctuation (RMSF) Profiles. The RMSF of Cα atoms was calculated over the 300ns molecular dynamics trajectories to quantify the local flexibility of each residue along the protein chain. (A) Overlays the profile for the WT and all five mutants, enabling a visual comparison of highly mobile regions (peaks) across the entire sequence (Residue Number on the X-axis). Other Panels (B): Top Sub-Panel: Compares the absolute profiles of the mutant (colored dashed line) against the WT (black solid line). Bottom Sub-Panel (ΔRMSF): Plots the differential fluctuation (RMSF_Mutant_ −RMSF_WT_). Positive peaks indicate segments of the chain that became more flexible due to the mutation, while negative valleys indicate regions that became stabilized or more rigid. Each differential plot includes the Wilcoxon Signed-Rank Test -value, comparing the overall distribution of the mutant’s RMSF values to the WT, with the conclusion confirming whether the global change in flexibility is statistically significant.

The RMSF for the atoms was calculated across the concatenated trajectories to identify localized regions of structural plasticity. The figure presents the residue-averaged RMSF for Chain A of the tetramer (Fig 7). However, we confirm that the single chain is a valid representation of the complex’s local dynamics, as shown by the comparison to the tetramer in S3 Fig. The combined RMSF plot (Fig 7A) shows that all mutants preserve the WT’s general fluctuation pattern, with the highest flexibility in key loop regions (e.g., residues 140–150 and 260–270). To rigorously quantify the differences between the systems, the Wilcoxon test was applied to the per-residue RMSF distributions (Mutant vs. WT), and the results were corrected for multiple testing using the strict Bonferroni method (α = 0.01).

The ΔRMSF plots (Fig 7B, showing RMSF_Mutant_ ─ RMSF_WT_) highlight the statistically significant (or non-significant) differences in local dynamics. The mutant E229S exhibits the most widespread and statistically significant increases in flexibility across the entire protein backbone. The major increases are found in the critical active site loops (140–150 and 260–270). The overall change is Highly Significant (P-value_Wilcoxon_= 7.66e^−12^), strongly correlating with its maximum global instability (high RMSD KDE mean). K207I displays a pattern of statistically significant increased flexibility concentrated in the flexible loop regions. This confirms that the global shift toward an expanded KDE state is mechanistically driven by localized structural loosening. The RMSF change is concluded as highly significant (P-value_Wilcoxon_= 1.6e^−12^). The G324T mutant shows a generally moderate, yet statistically robust increase in flexibility (ΔRMSF) compared to the WT. The statistical analysis also confirms this change is highly significant (P-value_Bonferroni_ ≈ 1.0e^−2^), indicating that the substitution causes a subtle, non-random dynamic loosening across the fold. In contrast to the others the RMSF analysis concludes that K207H mutant exhibits no statistically significant difference in local flexibility compared to the WT baseline. Although the RMSD KDE showed this mutant retains the WT’s bi-modal switching, the lack of significant RMSF change demonstrates that the inherent local dynamic profile remains largely unperturbed by the Histidine substitution. Similarly, the K207W mutant shows minimal ΔRMSF and is also statistically concluded as not significant (P-value_Wilcoxon_= 7.81e^−03^), failing to meet the Bonferroni corrected threshold).

To confirm the overall structural integrity of the biologically active state, the Radius of Gyration R_g_ of the entire tetrameric assembly C-α atoms was calculated over the 300 ns trajectory for all variants. As shown in S4 Fig, the R_g_ distributions for the Wild Type and all five mutants are tightly clustered, differing by less than 0.04 nm in their mean values. This demonstrates that the quaternary structure maintains its overall compactness and global stability throughout the simulation time, confirming the structural relevance of the model used. Given the verified global stability of the tetrameric assembly, which confirms that inter-subunit arrangements are robustly maintained, we proceeded to analyze the subtle, localized conformational changes within the individual catalytic unit (Chain A).

**Fig 8:**
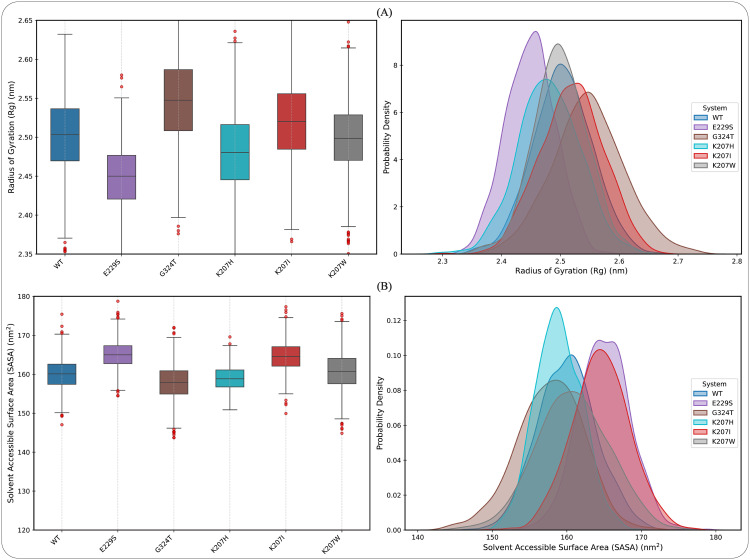
Ensemble distributions of size (R_g_) and solvent exposure (SASA). This figure presents ensemble properties of variants analyzed from molecular dynamics trajectories. Left panels feature box plots, with the central line indicating the median, box edges representing the interquartile range (IQR), and red circles marking outliers. Right panels display KDEs for each metric, illustrating the full conformational range, statistical mean (vertical dashed lines), and distinct shapes of conformational ensembles. The R_g_ (A) and SASA (B) plots demonstrate statistically significant shifts in ensemble properties for all mutants compared to the WT, confirming altered conformational landscapes. The precise median values and the statistical significance (*P*-values) for all systems are quantified and presented (Table 4).

The ensemble properties of the backbone structure were further analyzed using the R_g_, which measures global compactness, and the Solvent Accessible Surface Area (SASA), which quantifies the protein’s exposure to solvent. The distributions of these parameters for the ensembles are presented in Fig 8, and the statistical comparison using the Mann-Whitney U test against the WT is summarized in Table 3. The analysis reveals statistically significant shifts in the overall volume occupied by the protein ensemble for all mutants (***p < 0.001), confirming that every substitution perturbs the native packing of (Table 3). The distribution plots (Fig 8) show that the mutants fall into three distinct categories. Mutants G324T and K207I show median R_g_ values greater than the WT, representing a preference for structurally expanded conformations. Mutants E229S and K207H display median R_g_ values significantly lower than the WT, indicating that the ensemble samples conformations that are, on average, more tightly packed or internally compressed than the native WT structure. The K207W mutant exhibits a median R_g_ value (2.50 nm) that is structurally closest to the WT median (2.51 nm). Furthermore, we determined SASA fit for measuring the protein’s overall surface area exposed to the solvent, and a key indicator of tertiary fold stability and potential aggregation tendency. The analysis showed statistically significant changes for all mutants compared to the WT (***p < 0.001). These three mutants (E229S, K207I, and K207W) all show a significantly increased median SASA, meaning their ensembles expose a larger hydrophobic and/or polar surface area to the solvent. This is typical of structures that are less optimally packed or possess regions of unraveling. These two mutants (G324T and K207H) surprisingly show a significantly decreased median SASA, suggesting that their substitution leads to a folding configuration where the exposed surface area is minimized.

**Table 4:** Genetic diversity and selective pressure metrics of H5N1 neuraminidase (NA1) genes isolated from diverse hosts (2023–2024).

Metrics	Human	Cattle	Chicken	Turkey	Swan	Geese
**No of sequences**	26	353	270	56	125	171
**(S)**	397	328	501	153	280	468
**η**	460	343	0.9999	160	302	536
**(h)**	23	334	243	55	119	167
**(Hd)**	0.991	0.9996	0.06766	0.999	0.992	0.9995
**π**	0.10976	0.00357	0.03221	0.02007	0.01811	0.02666
**π (s)**	0.39662	0.01008	0.10426	0.06120	0.06120	0.08492
**π (a)**	0.02776	0.00175	0.01175	0.00845	0.00845	0.00998
**θη**	0.09036	0.03776	0.06766	0.0247	0.04069	0.06780
**θS**	0.07799	0.03611	0.05805	0.02362	0.03773	0.05920
**k**	146.422	5.037	45.024	28.294	24.884	36.864

The genetic diversity metrics were calculated as follows: S, the number of polymorphic sites; η, the total number of mutations; h, the number of haplotypes; Hd, the haplotype diversity; π, the nucleotide diversity; π(s), the synonymous nucleotide diversity; π(a), the non-synonymous nucleotide diversity; θη, Theta per site is estimated from Eta; θS, Theta per site is estimated from S and k, the average number of nucleotide differences.

### Free energy landscape analysis

**Fig 9:**
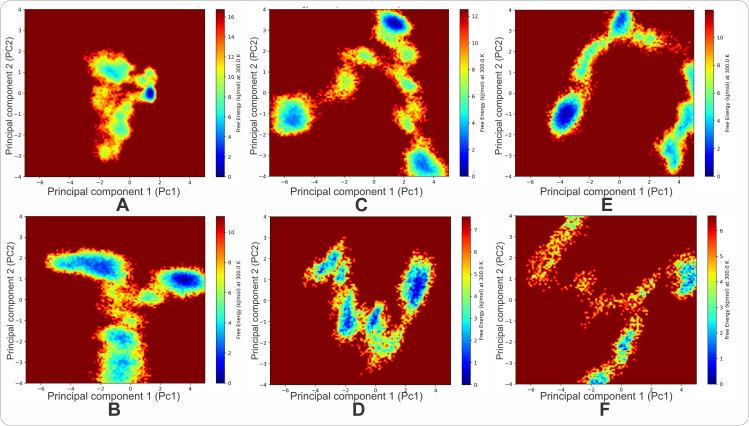
Conformational Free Energy Landscapes (FELs) of the NA1 WT and Variants. The FELs were constructed by projecting the 300 ns molecular dynamics trajectories onto the first two principal components (PC1 and PC2), which capture the maximum variance in the overall backbone motion. (A) The Wild-Type (WT) shows a bi-modal landscape with two connected deep minima (dynamic switching). (B) The K207W mutant exhibits a single, deep, rigid minimum (structural lock). (C) The E229S mutant displays a fragmented, shallow landscape indicative of maximum conformational entropy (disordered collapse). (D-F) show the landscapes for K207H (dynamic preservation), K207I (expanded disorder), and G324T (single-axis restriction), respectively.

Free Energy Landscapes (FELs) were constructed by projecting 300 ns trajectories onto the first two principal components (PC1 and PC2), which capture the majority of backbone motion variance. Blue basins indicate stable, frequently sampled conformations, while red/yellow regions represent high energy barriers or unsampled space. This analysis provides a detailed understanding of conformational dynamics across different protein variants. The Wild-Type (WT) FEL, serving as a baseline, reveals a dominant central basin and a distinct secondary minimum around *PC1 ≈ 1.5, PC2 ≈ 0*. This bi-modal landscape confirms that the native protein samples two principal conformational states connected by a low-energy pathway, consistent with observed bi-modal RMSD KDE and intrinsic malleability. In contrast, destabilizing mutants K207I and E229S exhibit highly altered, distributed landscapes; E229S shows a fragmented, shallow landscape with numerous small minima, indicative of high conformational entropy, while K207I presents a diffuse, fragmented pattern with widely spread minima, reflecting a profound disruption of native energy barriers. Fine-tuning specialists K207H and G324T demonstrate controlled modifications. K207H maintains a clear multi-basin landscape with a shifted energy profile, preserving dynamic switching. G324T, however, displays a stretched, linear landscape, indicating a concerted, single-axis dynamic motion and a loss of rotational flexibility. Finally, the K207W mutant shows a “structural locking” mechanism, with a single, deep, and well-defined minimum, effectively trapping the protein in a rigid conformational state by abolishing native dynamic switching due to high energy barriers Fig 9.

### Dynamic cross-correlation matrix (DCCM) analysis

**Fig 10:**
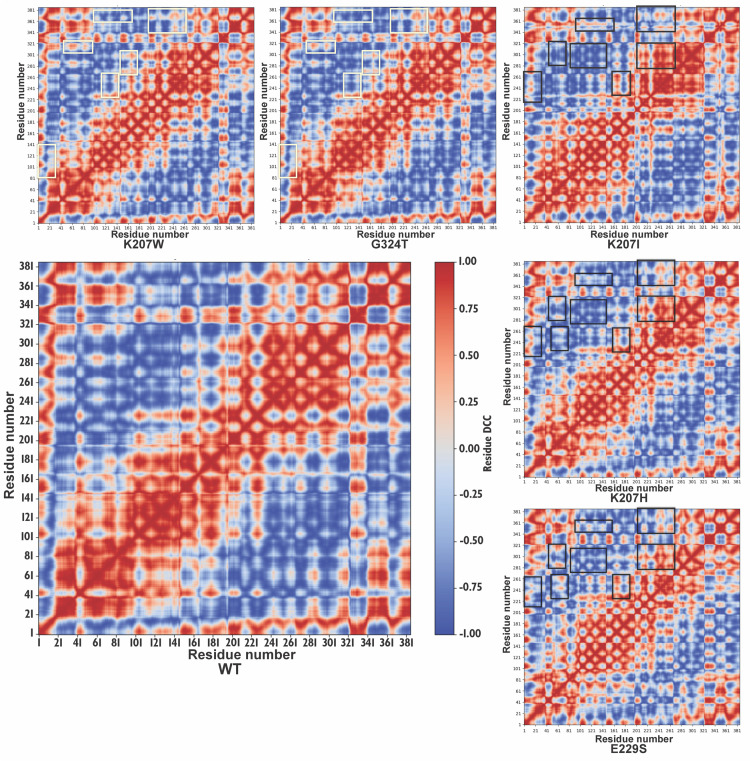
Dynamical Cross-Correlation Maps (DCCMs) of wild-type and family-specific position-based mutant NA1 catalytic domains. The DCCMs for the WT NA1 catalytic domain (specifically, Chain A) and its FSP-based mutant proteins, were derived from 100 ns of molecular dynamics simulation. These maps visualize correlated motions between residue pairs, with red indicating strong positive correlation (residues moving in the same direction) and blue indicating strong negative correlation (residues moving in opposite directions). White and black rectangular boxes highlight regions (e.g., residues 1-40, 140-180, 200-240, 300-380) where the most notable alterations in correlation patterns are observed in the mutants compared to WT. These maps provide atomic-level insights into how FSP mutations perturb the protein’s internal communication networks and conformational flexibility.

Dynamical Cross-Correlation Maps (DCCMs) were generated to provide atomic-level insights into the correlated motions between residues within the wild-type (WT) and mutant NA1 proteins (Fig 10). These maps use a color scale where red indicates strong positive correlation (residues moving in the same direction), blue indicates strong negative correlation (residues moving in opposite directions), and white/light blue indicates little to no linear correlation. The large matrix for the WT NA1 reveals extensive regions of strong positive correlation (red blocks along and off the diagonal), indicating concerted movements within and between structural sub-regions of the catalytic domain. The presence of discernible anti-correlated regions (blue blocks) also suggested inherent flexibility and allosteric communication pathways within the native protein. In contrast, the five smaller matrices for the mutant NA1 constructs (K207W, G324T, K207I, K207H, E229S) demonstrated distinct alterations in their correlation patterns compared to WT. Specifically, white and black rectangular boxes in Fig 6 highlight regions where the most notable changes in correlation patterns occurred, including residues 1–40, 140–180, 200–240, and 300–380. Each mutant exhibited unique changes in both positive and negative correlation blocks within these and other regions. These widespread changes in correlated motions suggest that mutations at the identified FSPs perturb the global dynamic landscape of NA1, influencing its internal communication and conformational flexibility.

### Genetic diversity and evolution of the H5N1 neuraminidase gene across multiple hosts

A comprehensive analysis of the H5N1 neuraminidase gene across six different hosts—chicken, turkey, geese, swan, human and cattle—revealed substantial genetic diversity on the basis of an examination of 26–353 sequences per host (Table 4). Analysis of polymorphic sites revealed variations ranging from 153 to 501 across hosts, with chickens showing the greatest number of polymorphic sites (501) and turkey showing the lowest (153). The haplotype diversity (Hd) was notably high across all hosts, ranging from 0.9910 in humans to 0.9996 in cattle, demonstrating considerable genetic variability in viral populations. Nucleotide diversity (π) varied significantly, with humans exhibiting the highest diversity at 0.10976 (10.98%), followed by geese at 0.08492 (8.49%) and swans at 0.05529 (5.53%). Further examination of nucleotide diversity highlighted differences in synonymous and nonsynonymous substitutions among the hosts. Cattle presented a synonymous nucleotide diversity (π(s)) of 0.01008 and a nonsynonymous nucleotide diversity (π(a)) of 0.00175, whereas chicken presented π(s)of 0.10426 and π(a) of 0.01175. The geese sequences presented π(s) and π(a) values of 0.08492 and 0.00998, respectively, and the human sequences presented the highest synonymous nucleotide diversity, with a π (s) value of 0.39662 and a π(a) value of 0.02776. The swan and turkey sequences presented π(s) values of 0.05529 and 0.06120, respectively. The average number of nucleotide differences ranged from 5.037 in cattle to 146.422 in humans, indicating significant genetic variation. These findings underscore the extensive genetic diversity and variation in viral genes across different hosts, offering crucial insights into the virus’s evolutionary dynamics and adaptive mechanisms of viruses.

The McDonald--Kreitman (MK) test analysis of a viral gene isolated from multiple hosts revealed distinct selection patterns. In humans and swans, the results indicated neutral selection, with a neutrality index (NI) of 0.761 and an alpha value of 0.239. The nonsignificant results of Fisher’s exact test (P_value > 0.05) and the G test (P_value > 0.05) further support these results. Geese displays evidence of purifying selection, characterized by an NI of 1.622 and an alpha value of -0.622. Conversely, chicken suggests positive selection, with an NI of 0.495 and an alpha value of 0.505, although the statistical tests are not significant (P_value > 0.05). Both cattle and turkey both exhibit strong signals of purifying selection. Cattle have an NI of 2.493 and an alpha value of -1.493, with significant Fisher’s exact test (P_value = 0.000751) and G test (P_value = 0.00062) results. Similarly, turkey has an NI of 2.591 and an alpha value of -1.591, along with significant Fisher’s exact test (P_value = 0.002370) and G test (P_value = 0.00176) results (Table 5).

**Table 5:** McDonald-Kreitman test results for H5N1 neuraminidase (NA1) gene sequences, indicating selection pressures.

Metric	Human	Cattle	Chicken	Turkey	Swan	Goose
**Fixed Synonymous**	53	69	6	83	53	32
**Polymorphic Synonymous**	209	173	333	98	209	296
**Fixed Nonsynonymous**	16	20	4	17	16	8
**Polymorphic Nonsynonymous**	48	125	110	52	48	120
**Neutrality Index (Ni)**	0.76	2.49	0.50	2.59	0.76	1.62
**Alpha Value**	0.24	-1.49	0.51	-1.59	0.24	-0.62
**Fisher’s Exact Test P-Value**	0.50	0.00	0.28	0.00	0.50	0.27
**G Test P-Value**	0.41	0.00	0.30	0.00	0.41	0.22
**Selection Type Indication**	Neutral	Purifying	Positive	Purifying	Neutral	Purifying

The McDonald-Kreitman test analysis is used to calculate the Neutrality Index (NI) and Alpha value, which estimates selection pressures. Statistical tests (Fisher’s Exact and G Test) assess the significance of these ratios, indicating whether selection is neutral, positive, or purifying.

### Host-associated phylogenetic analysis of the H5N1 neuraminidase gene

**Fig 11:**
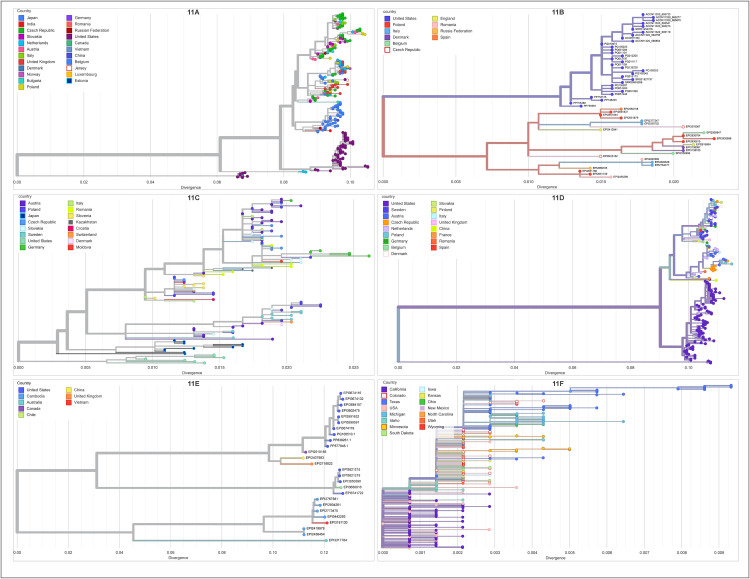
Host-associated phylogenetic analysis of H5N1 neuraminidase (NA1) gene sequences from six host species. This figure presents a comprehensive set of representative rooted phylogenetic trees of the H5N1 neuraminidase (NA1) gene, illustrating distinct patterns of genetic diversity across different hosts and geographical regions. The trees are displayed as follows: Panel (11A) displays the tree for Chicken isolates, Panel (11B) shows the tree for Turkey isolates, Panel (11C) shows the tree for Swan isolates, Panel (11D) shows the tree for Geese isolates, Panel (11E) shows the tree for Human isolates, and Panel (11F) shows the tree for Cattle isolates. For all panels, branch and node colors indicate the country of isolation as defined in the accompanying color legend, with the exception of Panel (C) for Cattle isolates, where node colors indicate the specific US state of isolation.

Host-associated phylogenetic analysis was performed on the NA1 gene of HPAI H5N1 viruses isolated from six primary host species (chicken, turkey, swan, geese, human, and cattle) across Europe, Asia, and the Americas. The resulting time-resolved phylogenetic trees (Fig 11) quantify patterns of genetic diversity, geographic structure, and evolutionary dynamics across these hosts. The NA1 sequences from chicken isolates (Fig 11A) exhibited high genetic diversity, showing a divergence range up to ~0.10. Multiple sequences originating from Europe (e.g., Germany, Netherlands, Poland) and Asia (Japan, India) intermingled across several major, deep clades, indicating high global transmission and intercontinental exchange in this host. In sharp contrast, turkey isolates (Fig 11B) displayed a highly constrained pattern of genetic diversity, with a maximum divergence of only ~0.02. European isolates (Italy, Spain, Romania) formed multiple distinct, short-branched clusters characterized by minimal internal divergence. Notably, United States isolates for turkey established a long, separate lineage basal to the main European clusters, indicating strong geographic separation. Swan isolates (Fig 11C) showed intermediate genetic diversity (~0.025 divergence), and their sequences segregated into two primary, deeply divergent clades. European sequences (Austria, Poland, Germany) dominated one clade, while a distinct lineage comprised sequences exclusively from Japan, demonstrating a strong pattern of geographic segregation within the swan population. Geese isolates (Fig 11D) demonstrated significant overall diversity (~0.10 divergence) coupled with strong geographic structure. European sequences (Belgium, Germany, Denmark) composed a large, dominant, and internally diverse clade, whereas North American isolates (United States) clustered distinctly in a highly diverged, separate basal lineage. Among the mammalian hosts, human H5N1 NA1 sequences (Fig 11E) presented the highest overall genetic diversity across all groups examined, with a divergence range extending to ~0.12. The sequences segregated primarily into distinct continental clusters: older, highly divergent clades formed by Asian sequences (e.g., China, Vietnam, Thailand), and more recent, separate evolutionary branches occupied by sequences from North America (USA, Canada) and Europe (UK, Netherlands, Spain). In striking contrast, the NA1 sequences from cattle isolates (Fig 11F) exhibited the least genetic diversity (~0.009 divergence). Nearly all sequences clustered together in a single, tight, highly resolved monophyletic group characterized by exceptionally short branch lengths. This highly conserved lineage sampled a wide geographic range across the United States (e.g., Texas, Kansas, Michigan, New Mexico), suggesting a recent and rapid dissemination event.

## Discussion

Integrating sequence and structural information remains paramount for a comprehensive understanding of the molecular mechanisms underlying biological specificity within protein families. While sequence analysis provides insights into conservation and divergence at the residue level, protein structure elucidates the spatial arrangement of key functional elements, revealing conserved interaction interfaces, binding pockets, and allosteric sites that may not be apparent from sequence alignments alone [30,31]. Furthermore, comparative structural analyses, alongside computational approaches, can illuminate subtle evolutionary adaptations and functional diversification within a family, offering a deeper understanding of their distinct roles. Here, our comparative structural analysis of the nine NA subtypes revealed a striking dichotomy: despite their high structural similarity, the sequences exhibited a complete interplay of conservation and diversity across subtypes. While neuraminidase subtypes maintain a conserved core structural function, the observed sequence diversity among them plays a crucial role in modulating functional adaptations. This sequence variability, particularly at FSPs, not only enables the enzyme to fine-tune interactions with different host factors and immune defenses [32–34] but also confers protein flexibility that facilitates the adaptation of H5N1 to new hosts. Thus, the inherent structural integrity of IAV neuraminidase subtypes ensures fundamental activity, whereas sequence diversity, especially at FSPs, drives altered protein dynamics, leading to the functional diversification which may be necessary for viral evolution and cross-species transmission.

Previous studies, [35,36] have grouped the nine NA subtypes into two broad categories. Interestingly, our phylogenetic analysis, which reconstructed the evolutionary history of the nine subtypes, yielded similar results, reinforcing the prevailing view. However, when we employed a graph theory approach, which focused on functional relationships between proteins, our analysis revealed a more nuanced classification, separating the nine subtypes into four distinct groups. The distinction between our phylogenetic findings and the graph theory results highlights the influence of methodology on classification outcomes. Phylogenetic tree construction relies on maximum parsimony or likelihood principles to reconstruct evolutionary histories, prioritizing overall sequence similarity, branch lengths, and node relationships. In contrast, the graph theory approach emphasizes the similarity of amino acid residues in specific columns, identifying clusters of proteins with similar functional characteristics. The disparity between our phylogenetic and graph theory findings underscores the importance of considering multiple classification methods. By integrating phylogenetic and functional perspectives, researchers can gain a more comprehensive understanding of protein evolution and function. Our results demonstrate the value of graph theory approaches in revealing subtle functional differences within protein families, which may not be apparent through phylogenetic analysis alone.

The rigorous analysis across RMSD, RMSF, R_g_, SASA, and FEL parameters reveals that single-point mutations in FSPs function as molecular switches that reprogram the enzyme’s dynamic landscape to suit distinct functional or evolutionary pressures. The WT NA1 structure establishes a dynamic baseline, existing as a highly sophisticated system optimized for conformational breadth, or ‘malleability.’ Furthermore, the bi-modal nature of its RMSD distributions, confirmed by the corresponding multi-basin topography of the FEL, validates the existence of a functionally critical dynamic switch—an intrinsic property that allows the enzyme to sample two primary low-energy states. This dynamic equilibrium is achieved while the enzyme maintains a highly ordered local structure, as indicated by non-significant RMSF for key loops, and preserves an optimized volume (R_g_ = 2.51 nm). Critically, the position K207 emerges as the master determinant of the enzyme’s dynamic fate. This residue exhibits the highest statistical significance in its ability to generate diametrically opposed outcomes upon substitution. The native Lysine serves as a dynamic nexus, providing the local flexibility required to facilitate the bi-modal WT landscape. When mutated, this single site dictates the enzyme’s dynamic specialization through three distinct mechanisms.

First, the Tryptophan substitution (K207W) imposes a structural rigidification, effectively acting as a structural lock. The resulting unimodal FEL and the non-significant change in RMSF confirm the abolition of the native dynamic switching mechanism, forcing the enzyme into a single, rigid conformational state that maintains a near-WT volume (R_g_ = 2.50 nm). The high energy barriers established by the bulky Tryptophan side chain dictate a fate of structural control. Second, the Histidine substitution (K207H) achieves dynamic preservation. This mutant preserves the enzyme’s core mechanism, resulting in a non-significant RMSF profile and an FEL that retains the multi-basin topography characteristic of the WT enzyme. This suggests a strategy of dynamic optimization, where the enzyme achieves its functional specialization while maintaining the essential conformational flexibility, albeit in a slightly more compact and sheltered state (R_g_ = 2.47 nm, decreased SASA). Third, the Isoleucine substitution (K207I) drives an expanded disorder and collapse. The highly significant RMSF values, coupled with the diffuse, fragmented FEL, confirm that the loss of the native Lysine side chain’s properties allows for the loosening and global expansion of local loops, resulting in an unstable, expanded ensemble (R_g_ = 2.53 nm). The functional diversity anchored at position K207 demonstrates that subtle side-chain differences at this pivotal residue can produce diametrically opposite dynamic outcomes, showcasing its essential role in dictating the enzyme’s evolutionary trajectory.

The remaining mutants further define the hierarchy of perturbation, acting as mechanisms of localized restriction rather than master switches. While K207 controls the *type* of dynamic specialization, the E229S mutant causes the largest magnitude of disorder, leading to catastrophic disorder. The loss of the E229 electrostatic anchor drives a structural failure defined by maximum global RMSF, increased SASA, and an FEL of maximum conformational entropy. The paradoxical decrease in R_g_ (compact 2.45 nm) confirms that this represents a disordered collapse, or a “compact molten globule,” where the structure has failed internally. The G324T mutant, conversely, imposes a Linear Restriction, introducing a specific kinetic constraint. The unique linear structure of its FEL demonstrates that this substitution forces the ensemble’s motion onto a single, dominant axis, abolishing complex dynamic rotation. This restriction results in an expanded R_g_ state that paradoxically becomes less exposed (decreased SASA).

The atomic-level underpinning of this dynamic specialization is further elucidated by the calculated DCCMs. Crucially, the DCCMs (Fig 10), also computed for a single representative chain (e.g., Chain A) from the tetramer simulations, provided atomic-level insights into how single-point mutations—Functional Specialization Points (FSPs)—modulate NA1 function and contribute to its evolutionary divergence. To address the necessity of analyzing the biologically active tetramer, we note that the RMSF profile of the single subunit (Chain A) shows strong congruence with the RMSF profile of the whole tetramer (S3 Fig), indicating that the intrinsic dynamic signature of the catalytic domain is robustly maintained within the quaternary structure. This congruence justifies the use of a single-chain DCCM to effectively isolate and analyze intra-monomeric network communication. The widespread alterations in correlated motions within mutant NA1 constructs, particularly evident in the white rectangular boxes highlighting regions such as residues 1–40, 140–180, 200–240, and 300–380, demonstrate that FSP mutations significantly re-wire the protein’s internal communication networks. These dynamic perturbations, occurring within and between structural sub-regions of the catalytic domain, extend beyond the local effects typically associated with the well-characterized 150-loop [7,37,38]. Such FSP-induced changes in conformational flexibility and allosteric coupling are inferred to be fundamental to the functional specialization observed across NA subtypes and the adaptive evolution of NA1 to diverse hosts. For instance, these altered dynamic patterns provide a mechanistic hypothesis for changes in substrate recognition, altered enzymatic efficiency, and modified susceptibility to antiviral inhibitors, all of which drive the functional diversity necessary for viral fitness and host range expansion. While the overall stoichiometric effect of the tetramer is the ultimate determinant of macroscopic enzymatic function (such as K_max_ or V_max_), the computational findings presented here offer vital atomic-level evidence regarding the *causal* dynamic perturbations that underpin these changes. This mechanistic understanding is essential for elucidating the precise underpinnings of NA1’s adaptive evolution and for guiding the rational design of targeted antiviral strategies.

The *NA1* of H5N1 exhibits substantial genetic diversity across host species, underpinning viral adaptation and potentially augmenting transmissibility and virulence. Notably, human infections display high within-host diversity—suggesting ongoing adaptive evolution with putative human-adapting mutations emerging during spillover events—while the range of polymorphic sites (153–501) highlights host-specific influences that drive antigenic drift and adaptation. Moreover, the exceptionally high haplotype diversity (0.9910–0.9996) underscores the virus’s robust capacity for mutation and recombination, facilitating rapid adaptation to new hosts and environments and predisposing H5N1 to sustained human-to-human transmission via immune evasion. Consistent with these findings, analysis of nucleotide diversity (π) reveals significant interhost variation: human isolates presented with the highest diversity (10.98%), followed by geese (8.49%) and swans (5.53%), whereas cattle (0.36%) and turkeys (2.01%) display comparatively lower variation. Although such genetic variability is pivotal in enabling adaptability, rapid viral evolution also hinges on host factors such as the immune response, population density, and coinfections. Overall, the extensive diversity within the *NA1* provides a versatile genetic reservoir from which advantageous mutations may arise, thereby promoting host adaptation and immune escape.

The McDonald--Kreitman test reveals heterogeneous selection pressures on the *NA1* gene across different hosts, reflecting the complex interplay between viral evolution and host-mediated forces. In humans and swans, neutral selection predominates—evidenced by a balanced ratio of nonsynonymous to synonymous substitutions [39]—suggesting that these hosts exert minimal evolutionary pressure on the gene. Conversely, in geese, cattle, and turkeys, strong purifying selection acts to eliminate deleterious alleles, thereby conserving essential functions [40]. Notably, chicken-derived isolates exhibited signs of positive selection, albeit with limited statistical support, indicating the potential fixation of advantageous mutations in response to specific environmental or host factors [41]. Together, these findings underscore the adaptive potential of the H5N1 *NA1* in diverse host contexts, highlighting the necessity of multifaceted analytical approaches to accurately predict viral emergence and inform strategic responses to zoonotic threats such as H5N1 [42,43].

The comparative phylogenetic analysis of the H5N1 NA1 gene across six host species reveals distinct host-associated patterns that dictate viral evolution, dispersal, and maintenance. Specifically, the high NA1 gene diversity observed in viruses isolated from chickens, geese, and swans, particularly within Asia, aligns with previous studies that highlight this region as a hotspot for Influenza A virus (IAV) genetic diversification and potential zoonotic emergence [44,45]. Chickens act as the primary reservoir and evolutionary “midwife.” High genetic diversity and intermingled continental clustering (Asian and European sequences) indicate sustained circulation and persistent spillover from wild birds and global trade, creating a large, mixed poultry viral pool.

The resulting deep divergence and highly fragmented NA1 structure serve as a clear signature of a hyper-reassortment environment. This accelerated segment exchange across key genes (NA and HA), driven by high-density farming, generates novel, virulent HPAI strains at a rate independent of natural evolution in wild birds. Geese and Swan exhibit patterns defined by deep geographic segregation (strong continental separation). This strong divergence confirms long-term genetic isolation, allowing independent H5N1 evolution to establish distinct, endemic lineages within these geographically separated wild bird populations over extended periods. Turkeys show a unique profile characterized by constrained diversity (~0.025) but multiple short, distinct European clusters. This topology supports repeated, independent spillover events leading to localized, rapid within-flock transmission. Turkeys are therefore frequent but localized recipient hosts rather than primary drivers of global NA1 lineage diversification.

The phylogenetic structure of mammalian isolates highlights critical differences in evolutionary timelines and transmissibility. Human isolates present the highest genetic divergence (~0.12) and deep continental separation. This confirms that human H5N1 infections remain sporadic, genetically diverse spillover events derived from multiple, distantly related avian lineages. This pattern reinforces the view that humans remain typical dead-end hosts, lacking widespread, sustained human-to-human transmission. In contrast, cattle isolates show the least genetic diversity (~0.009). The existence of a single, highly conserved monophyletic cluster with extremely short branch lengths across a vast geographic area suggests a **v**ry recent, rapid, and efficient epidemiological expansion of a single H5N1 lineage [15]. This phylogenetic signature implies a strong bottleneck upon host adaptation, followed by successful, high-volume animal-to-animal transmission. This finding provides critical evidence for a distinct host-adaptation event in cattle that necessitates intensified molecular and epidemiological surveillance.

While we observed varying degrees of diversity in chicken isolates across continents, likely reflecting differing exposure pressures, surveillance intensities, or evolutionary trajectories, the apparently greater *NA1* diversity in geese isolates from North America than in those from Europe and greater diversity in swan-derived H5N1 isolates from Asia, warrant cautious interpretation. These apparent differences can be heavily influenced by disparities in sampling size and surveillance efforts. Therefore, definitive conclusions regarding genuine biological distinctions in NA diversity across wild bird populations necessitate further research with rigorously comparable sample sizes. Perhaps of most importance, these observations reflect the significant role wild and migratory birds have on the introduction of new viral strains (or clades) in new environments and vice versa [46,47], making containment and disease management efforts an impossible task.

Our research culminates in a singular, critical imperative: to comprehensively understand the H5N1 influenza virus across two interconnected scales – the atomic resolution of its enzymatic machinery and the expansive panorama of its global emergence. The strategic synthesis of insights derived from these disparate scales not only illuminates the molecular Achilles’ heel of the virus but also underscores the profound urgency for a fundamental re-evaluation of global public health preparedness and response paradigms. Our investigation commenced by challenging conventional static models of enzyme function [48,49]. Through advanced molecular dynamics simulations, we redefined NA1 activity not as a rigid, predetermined lock-and-key interaction, but as an exquisitely calibrated dynamic landscape—a fluid architectural ensemble indispensable for its catalytic proficiency. Central to this dynamic milieu is the highly significant K207 residue, unequivocally identified as the enzyme’s pivotal conformational switch, dictating its evolutionary trajectory toward states of optimized rigidity, essential flexibility, or debilitating non-functional disorder. Critically, our structural analyses affirmed the invariant conservation of key family-specific positions defining the NA1 subtype across a diverse array of H5N1 isolates.

This intrinsic stability precludes major structural reconfigurations as the primary driver for rapid host adaptation events, such as recent zoonotic spillovers into bovine populations. Consequently, our focus now converges on the hypothesis that host tropism is not encoded within these static structural identity markers, but rather within subtle, yet profoundly impactful, dynamic alterations occurring at functionally regulatory sites. This leads directly to the ‘Paradox of K207’: despite the sequence conservation of the Lysine residue across diverse host species, our meticulous MD analysis definitively established the K207 position as the enzyme’s dynamic nexus. Employing targeted mutations as experimental probes, we unequivocally demonstrated that even minor perturbations to the biophysical characteristics at this singular, conserved site—whether by enforcing a rigid state or inducing detrimental disorder—result in the catastrophic collapse of the catalytic mechanism. Therefore, K207 is not a locus of sequence-level adaptation, but rather a fundamental dynamic vulnerability that evolutionary pressures must either meticulously preserve or finely tune. This profound insight immediately furnishes a compelling strategy for the development of next-generation antiviral compounds. By precisely targeting this ‘Nexus for Dynamic Ablation,’ a novel therapeutic agent could be engineered to stereo-specifically bind in proximity to K207, thereby neutralizing the essential bi-modal switching capacity of the wild-type Lysine. Such an intervention would effectively entrain the enzyme in a permanent, non-functional conformation, achieving an irreversible cessation of its enzymatic activity.

While our molecular elucidation provides an ultimate therapeutic target, the concurrent phylogenetic analysis of the NA1 gene provides the compelling mandate for its expedited clinical translation and deployment. The stark juxtaposition of sustained, high-density evolutionary pressures observed within domestic poultry populations against the deeply divergent and highly scattered genetic profiles of human isolates yields a critical epidemiological lesson: the principal threat is not merely the stochastic, isolated emergence of H5N1, but rather the cumulative probability risk intrinsically embedded within every zoonotic spillover event. The pervasive genetic diversity observed among viruses isolated from sporadic human cases is not indicative of viral quiescence; conversely, it constitutes compelling evidence of persistent, heterogeneous evolutionary selection. This recurrent pattern confirms that spillover events are both frequent and genetically distinct, signifying repeated and varied exposures of the virus to the human host environment. With each successive exposure, the statistical probability of acquiring the requisite ensemble of pandemic-enabling mutations—including, but not limited to, receptor binding shifts and enhanced polymerase efficiency—is demonstrably augmented.

## Conclusion

The persistent and multifaceted threat of H5N1 adaptation necessitates a paradigm shift from reactive containment to proactive risk mitigation. This reorientation hinges on leveraging the genetic diversity in dead-end hosts as a crucial leading indicator of pandemic potential, thereby fundamentally transforming global surveillance efforts. Our molecular understanding of the K207 dynamic switch presents a high-value therapeutic target, while phylogenetic data underscores the urgent need for its rapid development and deployment. This strategic approach mandates two critical actions for ensuring adequate lead time: firstly, intensified global surveillance under the *One Health* framework, encompassing continuous and comprehensive genomic monitoring across avian, poultry, and human isolates. Secondly, aggressive investment in predictive tools, such as bioinformatic and structural models, is essential to seamlessly link genomic data with molecular vulnerabilities like K207. This integration will enable molecular forecasting, allowing for the instantaneous identification of high-risk strains and the implementation of swift, preemptive control measures. By unifying atomic and global perspectives, this strategy represents the future of pandemic preparedness, where molecular vulnerability informs predictive epidemiology.

## Materials and methods

### Structural alignment and comparative analysis of neuraminidase subtypes

To prepare a comprehensive dataset for family-specific position identification, nine high-resolution neuraminidase crystal structures were initially superimposed using the MatchMaker algorithm in ChimeraX for precise core structural alignment [50]. Visualizations were generated via Chimera and annotated with CorelDRAW X24. For structure-guided multiple sequence alignment, prealigned protein coordinates were exported for MUSTGUSEAL (Mode 2), which incorporates external alignments [51]. MUSTGUSEAL queried TrEMBL and UniProtKB/Swiss-Prot databases [52,53] using each NA subtype as a query, retrieving up to 500 sequences per subtype [54]. This dataset was rigorously curated by filtering out redundant sequences (>95% identity), eliminating outliers (sequence length deviations >±20% relative to query), and removing entries with a bit score per column threshold below 0.5. Filtered sequences within each subtype were realigned via MAFFT [55]. Finally, MUSTGUSEAL employed the NA1 protein structure (PDB ID: 2HTY) as a structural scaffold to generate a comprehensive multiple sequence alignment encompassing evolutionarily remote neuraminidase homologs.

### Phylogenetic construction of neuraminidase subtypes

A phylogenetic tree was constructed from the MUSTGUSEAL-generated structure-guided sequence alignment using a custom workflow [56]. To optimize accuracy, the alignment was curated via BMGE [57] to trim artifactual regions and focus on informative sites. Tree inference was performed with PhyML + SMS version 3.0 [58] due to the large dataset, employing the AIC statistical criterion for model selection, subtree pruning and regraphing methods for topology search, and SH-like aLRT for branch support. The resulting phylogenetic tree was visualized via iTOL version 4 in unrooted display mode [59], with final annotation conducted in CorelDRAW version 24.

### Classification of protein subfamilies and determination of family-specific positions

To classify sequences from the multiple sequence alignment of neuraminidase homologs into subfamilies, a graph theory approach implemented in ZEBRA2 was employed. In this framework, each sequence is a node, with edges established based on similarity quantified by a three-dimensional matrix. An iterative application of a similarity threshold connected sequences into clusters, systematically pruning low-connectivity nodes until the largest connected component achieved an edge network density of at least 70%. Clusters with fewer than three sequences were discarded, and the process continued until no new components emerged, yielding a robust classification into distinct subfamilies. Leveraging ZEBRA2’s scoring function, FSPs were identified by assessing the correlation between amino acid types and functional subfamilies. This function integrates relative entropy and a sum-of-pairs term considering physicochemical similarity, resulting in a specificity score (Si) from 0–1 (higher values denote higher specificity) [16]. The formula incorporates frequency terms: *q*_*i*_ (*AB,G*) (frequency of amino acid pair AB in subfamily G of column i), *q*_*i*_ (A) (frequency of residue A in column i), and *q*_*i*_ (*A,G*) (frequency of residue A within subfamily G of column i). Key variables include N (total sequences), *N*_*G*_ (sequences in subfamily G), *n*_*G*_ (total subfamilies), and M (A, B) (normalized physicochemical similarity between A and B). FSPs potentially driving NA1 functional diversity and host adaptability were identified by analyzing specificity scores. Scores were ranked by statistical significance, validated via random permutations, and correlated with shuffled scores via linear fit. The resulting Z-score, calculated from *n* random scores (S_*i*_^*rnd*^), served as a specificity estimate for each alignment column (higher values indicate higher specificity). Neighboring residues within 4Å in the 2HTY structure provided structural context for these estimates, enabling identification of key residues contributing to NA1 functional diversity and H5N1 host adaptation.

### Molecular dynamics simulation

To investigate the dynamic flexibility and mutation-induced structural perturbations of NA1, molecular dynamics simulations were performed on an H5N1 neuraminidase crystallographic model (apo 2HTY). Four chains (A–D) were selected for analysis. To assess the impact of FSPs on dynamics, five mutations (K207H, K207I, K207W, E229S, and G324T) were introduced into both structures via Wizard Mutagenesis in PyMOL based on the top three statistically significant FSPs. Critical stability factors, including water molecules and Ca² ⁺ ions present in 2HTY, were preserved. Simulations were conducted with GROMACS v2024.2 using the CHARMM36 force field [60], with disulfide bonds and protonation states assigned via CHARMM-GUI [61]. The TIP3P water model [62] was used for system solvation in a cubic box with a 1 nm buffer, and 20 mM NaCl was added for electrostatic neutrality [7]. Energy minimization was performed via the steepest descent algorithm (50,000 steps, extended to 100,000 for G324T), followed by a two-phase equilibration (100 ps under NVT and 100 ps under NPT conditions). Electrostatic interactions were computed via the particle mesh-Ewald method [63] with a 1.0 nm cutoff, while bonds were constrained via LINCS [64]. Temperature was maintained at 300 K via the V-rescale thermostat, and pressure at 1 bar via the Parrinello-Rahman barostat [65]. Production runs were executed in three independent 100 ns replicates for both native and mutant NA1 constructs, yielding a robust dataset to elucidate the mechanistic basis of NA1 conformational dynamics and its modulation by specific mutations.

#### Analysis of conformational dynamics and trajectory metrics.

For post-MDS analysis, trajectory files were refined to address periodic boundary conditions and molecular positioning. The three production replicates for each system were concatenated using utilities to form a single, aggregate trajectory, which served as the ensemble for all subsequent analysis. To ensure that the calculation measured internal conformational changes only, isolating them from mere rigid-body movement (translation and rotation) of the protein, the aggregate trajectories were aligned (fitted) to the starting minimized structure. This crucial alignment, which effectively removes rigid-body motion, was performed using the utility trjconv with the rot+trans option applied to the Cα backbone atoms. The refined and fitted trajectories were then used to generate a suite of metrics using utilities, including: Root Mean Square Deviation (RMSD), Root Mean Square Fluctuation (RMSF), Radius of Gyration (R_g_) and Solvent Accessible Surface Area (SASA).

The equilibrium ensemble for RMSD, R_g_, and SASA was visualized by generating the Probability Distribution Function (PDF) for each system (WT and mutants). This was achieved using a Kernel Density Estimate (KDE) methodology, which produces a high-resolution, smooth curve of the conformational space sampled. Two distinct non-parametric statistical tests were employed to rigorously quantify the differences between the and mutant systems. To test for statistically significant shifts in the central tendency (mean/median) of the RMSD, R_g_, and SASA distributions, the Mann-Whitney *U* test was performed. Each mutant’s distribution was compared pair-wise against the distribution for each metric.

The Wilcoxon Signed-Rank Test was used to test for statistically significant differences in the local flexibility profile. his test compared the distribution of Cα RMSF values across all residues of the target protein chain (Chain A) for each mutant against the corresponding WT chain. To correct for the potential accumulation of Type I errors arising from the multiple comparisons inherent in the RMSF profile analysis (comparing about 385 individual residues), the *P*-values derived from the Wilcoxon Signed-Rank Test were adjusted using the Bonferroni correction method. All trajectory calculations were performed using the GROMACS package. Subsequent data processing, statistical analysis (Mann-Whitney *U* test, Wilcoxon Signed-Rank Test, and Bonferroni correction), and high-resolution figure generation were conducted using a custom script developed in Python 3.10 relying on the following key libraries: NumPy (for array manipulation and calculation), SciPy.stats (for statistical testing), and Matplotlib/Seaborn (for KDE plot visualization and rendering).

#### Principal component analysis and generation of the free energy landscape.

To characterize the conformational space of the wild-type and mutant proteins, free energy landscapes (FELs) were generated from molecular dynamic trajectories. Principal component analysis (PCA) was performed on the backbone atoms of the proteins via the GROMACS tool gmx_mpi covar [66], calculating the covariance matrix of atomic fluctuations over the initial 100 ns. The trajectory was then projected onto the first two principal components (PC1 and PC2) using the gmx_mpi algorithm [66], capturing the largest collective motions within the 0–100 ns timeframe. The resulting two-dimensional projection data were used to construct the FEL via a Python script employing NumPy [67] and Matplotlib [68] libraries. A 2D histogram of PC1 and PC2 values was generated (0.1 units bin size, -4 to 4.1 range), with a small value added to avoid logarithmic singularities. The FEL was calculated as previously described [69,70], where F(PC1,PC2)=−kB TlnP(PC1,PC2), with P(PC1,PC2) being the probability distribution approximated by the normalized 2D histogram. The FEL was visualized as a contour plot via Matplotlib with a ‘jet’ colormap, where lower free energy regions (deeper blue) correspond to more frequently visited and stable conformations. This method allows for a detailed assessment of protein flexibility beyond traditional metrics.

#### Analysis of correlated residue motions via dynamical cross-correlation map (DCCM).

To investigate correlated residue motions, the Dynamical Cross-Correlation Map (DCCM) technique was employed via the MDAnalysis Python library [71]. This method quantifies movement relationships between residue pairs by analyzing positional fluctuations [72]. All C-alpha (Cα) atoms in chain B of 2HTY were selected to represent residue positions. The trajectory was centered at each time step based on the center of mass of the Cα atoms to eliminate overall translational motion. A data matrix (X) of size (3N × T) (N = number of residues, T = number of frames) was constructed from the Cartesian coordinates of the Cα atoms. From this, the covariance matrix (C) was calculated. The covariance between residues i and j, denoted covij, was obtained by averaging the trace of the (3 × 3) covariance submatrix. The variance for each residue i, denoted vari, was computed. The normalized cross-correlation coefficient (Dij) between residues i and j was calculated as described previously [73]. The resulting DCCM provides a matrix of these coefficients (ranging from -1 to +1), indicating the extent and direction of correlative motion. Values near +1 signify strong positive correlation, -1 strong negative correlation, and 0 little linear correlation. The magnitude of Dij reflects coupling strength. Residue pairs with zero variance were assigned a correlation value of zero. The DCCM matrix (D) was visualized as a heatmap via Matplotlib [68].

### *NA1* genetic diversity and adaptive evolution analysis

To investigate the genetic diversity and evolutionary dynamics of the *NA1* gene in H5N1 variants and clarify its adaptability across multiple hosts, *NA1* gene sequences (segment 6) deposited into Global Initiative on Sharing All Influenza Data (GISAID) [74] and Bacterial and Viral Bioinformatics Resource Center (BV-BRC) [75] databases between January 1, 2023 to December 31, 2024 were retrieved. This dataset included sequences from chickens (893), turkeys (176), geese (246), swans, humans (93), and cattle (600). Data quality was ensured via a custom in-house Python script that rigorously filtered out incomplete, invalid, and redundant entries. Host-specific multiple sequence alignments were generated via the MAFFT algorithm and refined via BMGE trimming. Maximum likelihood phylogenetic trees were inferred with RAxML (bootstrapped at 1000 replicates) to obtain robust evolutionary reconstructions [76]. The resulting phylogenetic trees and associated metadata were then processed using the Augur pipeline to annotate the tree with time and location data, estimate evolutionary rates, and infer ancestral states. Finally, the processed data was visualized interactively using Auspice, the web-based visualization tool for the Nextstrain platform, to explore the spatial and temporal dynamics of the host-specific clades. DNA polymorphisms, including measures of synonymous and nonsynonymous substitutions, were subsequently assessed using DnaSP version 6 on these curated alignments [77]. Furthermore, the McDonald-Kreitman (MK) test was applied by partitioning the H5N1 *NA1* sequences into test groups for each host and comparing them against an H1N1 *NA1* outgroup to detect signatures of adaptive evolution, thereby revealing host-specific selective dynamics in the H5N1 *NA1*. Translated *NA1* sequences from H5N1 isolates across all hosts were downloaded from the respective databases, and subsequent sequence alignment facilitated the examination of the most statistically significant FSPs for host-specific mutations.

## Supporting information

S1 Fig Root Mean Square Deviation (RMSD) Trajectories. Time evolution of the Cα-backbone Root Mean Square Deviation (RMSD) over 100 nanoseconds (ns) of molecular dynamics simulation for the Wild Type (WT) protein and five mutant systems (K207W, E229S, K207H, K207I, G324T). Each panel displays the RMSD relative to the minimized starting structure (t = 0) for three independent replicate simulations (REP1, REP2, and REP3), demonstrating the stability and convergence of each system’s trajectory. RMSD is reported in nanometers (nm).
(TIF)

S2 Fig Probability Distribution Functions (PDF) of Backbone RMSD for Each Variant. These panels display the individual PDF of the backbone RMSD ensembles over the entire simulation. This serves as a detailed breakdown of the combined distribution shown in the main Fig 6, highlighting the specific shape and primary peak location (dashed red line) for each and mutant system.
(TIF)

S3 Fig Comparative RMSF per residue for neuraminidase systems. The figure displays the RMSF values calculated per residue (residues 1–385) for the A-chain of Neuraminidase across 300 nanoseconds of molecular dynamics simulation for the Wild Type (WT) and five different single-point mutants (E229S, G324T, K207H, K207I, and K207W). This comparison evaluates the influence of the oligomeric state (tetramer vs. monomer) on the intrinsic flexibility of the enzyme domain. The RMSF values are plotted against the residue index on the X-axis, ranging from 1 to 385. The Y-axis represents the RMSF in (nm), which quantifies the average movement or fluctuation of each residue’s C-α atom relative to its average position throughout the 300 ns simulation. Elevated RMSF values signify increased local flexibility or conformational disorder within specific regions of the enzyme. A solid blue line depicts the RMSF profile of Chain A when simulated as part of a full functional tetramer, comprising four chains (A, B, C, and D). In contrast, a dashed orange line illustrates the RMSF profile of Chain A.
(TIF)

S4 Fig Comparative Radius of Gyration (R_g_) Distribution of Neuraminidase Systems. The figure presents the distribution of the R_g_ values, calculated for the Cα atoms of the Neuraminidase tetramer over 300 nanoseconds of molecular dynamics simulation for the Wild Type (WT) and five single-point mutants (E229S, G324T, K207H, K207I, and K207W). The R_g_ is a measure of the protein’s compactness, where a smaller R_g_ indicates a more tightly packed conformation and a larger R_g_ suggests a more extended or unfolded structure. The central box encompasses the Interquartile Range (IQR) of the R_g_ values, spanning from the 25th percentile (Q1) to the 75th percentile (Q3). The horizontal line running through the center of the box marks the median, or 50th percentile, of the distribution. Vertical lines, known as whiskers, extend from the box to the most extreme data points that remain within 1.5 X IQR of the box boundaries. Individual data points falling outside these whiskers, represented by small diamonds, are designated as statistical outliers. Crucially, the Red Diamond overlaid on each box explicitly indicates the calculated Mean R_g_ for that specific simulation system. Collectively, the vertical spread of the box and whiskers serves as a visual indicator of the variability and conformational stability of the protein’s global structure throughout the molecular dynamics simulation time.
(TIF)
