# Supplementary figures and images for: Structural analysis of antigenic variation and adaptive evolution of the H5N1 neuraminidase gene
Source: PLoS Comput Biol. 2026 Jan 16;22(1):e1013903. doi: 10.1371/journal.pcbi.1013903 (PMC12826504; doi:10.1371/journal.pcbi.1013903)

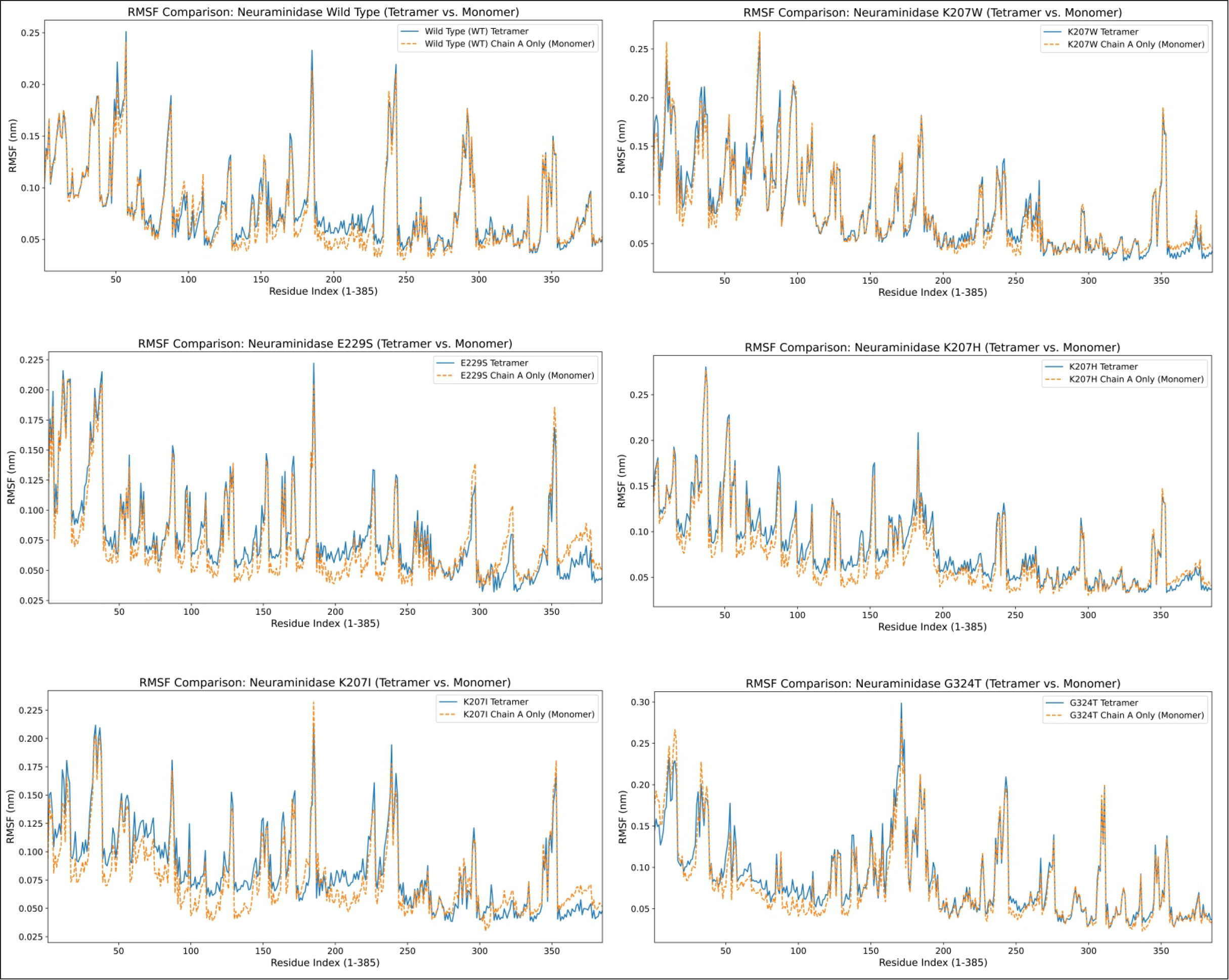

Supplement: S3 Fig — The figure displays the RMSF values calculated per residue (residues 1–385) for the A-chain of Neuraminidase across 300 nanoseconds of molecular dynamics simulation for the Wild Type (WT) and five different single-point mutants (E229S, G324T, K207H, K207I, and K207W). This comparison evaluates the influence of the oligomeric state (tetramer vs. monomer) on the intrinsic flexibility of the enzyme domain. The RMSF values are plotted against the residue index on the X-axis, ranging from 1 to 385. The Y-axis represents the RMSF in (nm), which quantifies the average movement or fluctuation of each residue’s C-α atom relative to its average position throughout the 300 ns simulation. Elevated RMSF values signify increased local flexibility or conformational disorder within specific regions of the enzyme. A solid blue line depicts the RMSF profile of Chain A when simulated as part of a full functional tetramer, comprising four chains (A, B, C, and D). In contrast, a dashed orange line illustrates the RMSF profile of Chain A. (TIF) [file pcbi.1013903.s003.tif]

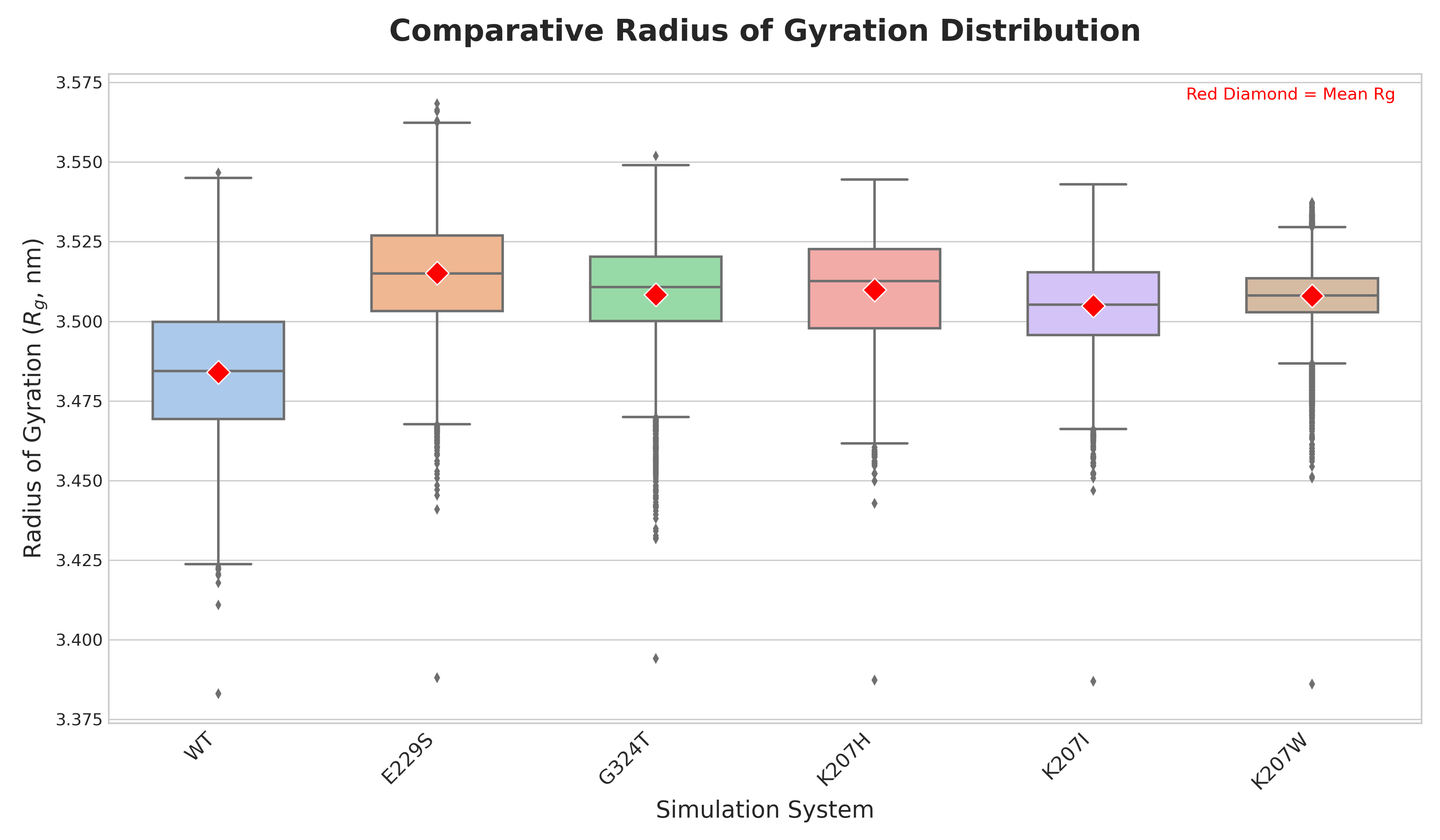

Supplement: S4 Fig — The figure presents the distribution of the Rg values, calculated for the Cα atoms of the Neuraminidase tetramer over 300 nanoseconds of molecular dynamics simulation for the Wild Type (WT) and five single-point mutants (E229S, G324T, K207H, K207I, and K207W). The Rg is a measure of the protein’s compactness, where a smaller Rg indicates a more tightly packed conformation and a larger Rg suggests a more extended or unfolded structure. The central box encompasses the Interquartile Range (IQR) of the Rg values, spanning from the 25th percentile (Q1) to the 75th percentile (Q3). The horizontal line running through the center of the box marks the median, or 50th percentile, of the distribution. Vertical lines, known as whiskers, extend from the box to the most extreme data points that remain within 1.5 X IQR of the box boundaries. Individual data points falling outside these whiskers, represented by small diamonds, are designated as statistical outliers. Crucially, the Red Diamond overlaid on each box explicitly indicates the calculated Mean Rg for that specific simulation system. Collectively, the vertical spread of the box and whiskers serves as a visual indicator of the variability and conformational stability of the protein’s global structure throughout the molecular dynamics simulation time. (TIF) [file pcbi.1013903.s004.tif]
